# Insect Pathogenic Bacteria in Integrated Pest Management

**DOI:** 10.3390/insects6020352

**Published:** 2015-04-14

**Authors:** Luca Ruiu

**Affiliations:** Dipartimento di Agraria, Sezione di Patologia Vegetale ed Entomologia, University of Sassari, Via E. de Nicola, 07100 Sassari, Italy; E-Mail: lucaruiu@uniss.it; Tel./Fax: +39-079-229326

**Keywords:** IPM, biopesticide, biological control, entomopathogen, *Bacillus thuringiensis*, *Photorhabdus*, *Xenorhabdus*, *Serratia*, *Streptomyces*, Spinosad

## Abstract

The scientific community working in the field of insect pathology is experiencing an increasing academic and industrial interest in the discovery and development of new bioinsecticides as environmentally friendly pest control tools to be integrated, in combination or rotation, with chemicals in pest management programs. In this scientific context, market data report a significant growth of the biopesticide segment. Acquisition of new technologies by multinational Ag-tech companies is the center of the present industrial environment. This trend is in line with the requirements of new regulations on Integrated Pest Management. After a few decades of research on microbial pest management dominated by *Bacillus thuringiensis* (*Bt*), novel bacterial species with innovative modes of action are being discovered and developed into new products. Significant cases include the entomopathogenic nematode symbionts *Photorhabdus* spp. and *Xenorhabdus* spp., *Serratia* species, *Yersinia entomophaga*, *Pseudomonas entomophila*, and the recently discovered Betaproteobacteria species *Burkholderia* spp. and *Chromobacterium* spp. Lastly, Actinobacteria species like *Streptomyces* spp*.* and *Saccharopolyspora* spp. have gained high commercial interest for the production of a variety of metabolites acting as potent insecticides. With the aim to give a timely picture of the cutting-edge advancements in this renewed research field, different representative cases are reported and discussed.

## 1. Introduction

Bacteria are widespread in the environment and they have evolved a variety of interactions with insects including essential symbiosis [1]. While many bacterial species inhabit bodies of insects establishing different levels of mutualistic relationships, only a limited number of them behave as insect pathogens. The latter have evolved a multiplicity of strategies to invade the host, to overcome its immune responses, to infect and to kill it. The mechanisms leading to these kinds of interactions are presumed to have ancient origin and to have developed throughout a long co-evolution process [2]. In line with this concept, a variety of insecticidal toxins produced by certain spore forming entomopathogenic bacteria, have a similar structure and mode of action. This is the case for protein toxins produced by *Bacillus thuringiensis* Berliner (*Bt*) and localized in parasporal bodies [3]. These toxins are normally very specific to a limited range of targets, while in other cases bacteria produce metabolites that show a broader insecticidal spectrum.

Important information to understand the molecular mechanisms involved in diverse pathogen-host interactions are being produced as a result of modern “omic” studies. However, many aspects are still unrevealed and after few decades of microbial pest management dominated by *B. thuringiensis*, novel bacterial species with innovative modes of action have been discovered and formulated as new biopesticidal products [4].

The entomopathogenic bacteria domain has traditionally been well represented by members of the *Bacillaceae* family, such as *B. thuringiensis*, *Lysinibacillus sphaericus* (Meyer & Neide) Ahmed *et al.*, *Paenibacillus* spp. and *Brevibacillus laterosporus* Laubach*.* The entomopathogens belonging to the Gammaproteobacteria class, which includes the entomopathogenic nematode symbionts *Photorhabdus* spp. and *Xenorhabdus* spp., *Serratia* species, *Yersinia entomophaga* Hurst and *Pseudomonas entomophila* (Mulet *et al.*), are also important.

More recent is the discovery of Betaproteobacteria species that show broad-spectrum insecticidal properties. This group includes specific strains of *Burkholderia* spp. and *Chromobacterium* spp. Lastly, certain Actinobacteria species have gained high scientific and commercial interest in relation to the production of a variety of metabolites acting as potent insecticides. This is the case for *Streptomyces* and *Saccharopolyspora* species.

As a result of continuous industrial and academic screening activities, the discovery of new bacterial species and insecticidal metabolites is expected in the near future [5,6]. This trend is also the result of modern legislative frameworks fostering the use of bioinsecticides in Integrated Pest Management (IPM) programs.

The purpose of the present review is to give an overall and concise picture of the knowledge advancements of insect pathogenic bacteria and of their use as bio-insecticidal products for IPM.

## 2. Insect Pathogenic Bacteria

### 2.1. Bacillaceae

#### 2.1.1. *Bacillus thuringiensis*

*Bacillus thuringiensis* (*Bt*) is the most studied entomopathogenic species and some of its crystal producing strains have certainly represented the main active substances used for the microbial pest management during the last decades [7]. The pathogenic action of this bacterium normally occurs after ingestion of spores and crystalline inclusions containing insecticidal δ-endotoxins that specifically interact with receptors in the insect midgut epithelial cells [8]. It is largely demonstrated that these toxins, mostly represented by Cry proteins, after being solubilized and activated in the insect midgut, act through a pore-forming mechanism determining the disruption of natural cell membrane permeability, with consequent cell lysis followed by gut paralysis and death [9].

Most commercially available formulations are based on spore-crystal mixtures with effectiveness against different pest species. *B. thuringiensis* subsp. *kurstaki* (*Btk*) is generally used against young Lepidopteran larvae and includes different strains with significant commercial interest like HD-1, SA-11, SA-12, PB 54, ABTS-351 and EG2348. Strains of *B. thuringiensis* subsp. *aizawai* (*Bta*) (*i.e.*, ABTS-1857) are also used against armyworms and diamondback moth larvae. Besides, strains belonging to the subsp. *israelensis* (*Bti*) and *tenebrionis* (*Btt*) have been employed for the management of mosquitoes and simulids, and against coleoptera, respectively [10]. Continuous research activities have led to the isolation of many strains and to the discovery and characterization of various *Bt* insecticidal toxins produced in different bacterial stages (*i.e.*, Cyt, VIP) [3]. The insecticidal potential of bacterial strains closely related to *B. thuringiensis* has also been demonstrated [11].

Additionally, the integration of *cry* genes into genetically modified plants has been successfully implemented to confer resistance to specific crop pests.

#### 2.1.2. *Lysinibacillus sphaericus*

Entomopathogenic strains belonging to the *L. sphaericus* (formerly *Bacillus sphaericus*) species group are featured by the production of spherical endospores closely associated with parasporal crystals containing an equimolar ratio of binary protein toxins (BinA and BinB) [12]. The insecticidal mode of action includes damages to the microvillar epithelial cells in the midgut comparable to the ones known for *B. thuringiensis* [13]. In addition, vegetative cells of certain strains produce mosquitocidal toxins (Mtx proteins).

The main targets of commercial formulations based on *L. sphaericus* strains are mosquitoes, blackflies and non-biting midges.

#### 2.1.3. *Paenibacillus* spp.

The genus *Paenibacillus* includes different species showing pathogenicity against insects like the causative agent of the honeybee disease American Foulbrood (AFB), *P. larvae* subsp. *larvae* [14].

On the other hand, the spore-formers *P. popilliae* (Dutky) Pettersson *et al.* and *P. lentimorbus* (Dutky) Pettersson *et al.* are the causal agents of milky disease in phytophagous coleopteran larvae. The production of parasporal inclusions within the sporangial cells has been observed in *P. popilliae*, even if they are not directly responsible for the insecticidal action. However, homology between a 80 kDa parasporal protein this species produces and *Bt* Cry toxins has been demonstrated [15]. After spores are ingested by the host, they germinate in the midgut. The following pathogenicity seems to be in relation to the septicemia caused by vegetative cells.

#### 2.1.4. *Brevibacillus laterosporus*

*Brevibacillus* (former *Bacillus*) *laterosporus* is a pathogen of invertebrates and a broad spectrum antimicrobial species [16]. During sporulation it produces a typical canoe-shaped parasporal body (CSPB) firmly associated with the spore coat, which gives this species a unique morphological feature. The insecticidal action of different *B. laterosporus* strains has been reported against insects in different orders, including Coleoptera, Lepidoptera and Diptera, and against mollusks, nematodes, phytopathogenic bacteria and fungi. In relation to its antifungal and antibacterial properties, due to the production of antibiotics, it has also found use in medicine.

The whole genome of *B. laterosporus* has recently been published [17,18], which reveals the potential to produce different toxins.

Certain strains showing toxicity against the corn rootworms (*Diabrotrica* spp.) and other coleopteran larvae, produce insecticidal secreted proteins (ISPs) that act as binary toxins in the insect midgut and have high homology with *B. thuringiensis* vegetative insecticidal proteins (VIPs) [19].

Specific strains toxic to mosquitoes produce parasporal inclusion bodies reminiscent of those produced by *B. thuringiensis*. These bodies contain proteins and their implication in the mosquitocidal action has been reported [20]. Spores of a strain lacking parasporal crystals are highly toxic to the house fly *Musca domestica* L., and the mode of action implies histopathological changes in the midgut with disruption of the microvillar epithelium [21,22].

### 2.2. Clostridiaceae

#### *Clostridium bifermentans* 

A strain of *C. bifermentans* (Weinberg and Séguin) Bergey *et al.* serovar *malaysia* (C.b.m.), isolated in Malaysia, shows high toxicity against mosquitoes and black flies. During sporulation, this bacterium produces three major proteins involved in the insecticidal action [23]. These include the mosquitocidal protein Cbm71 showing homology to *B. thuringiensis* delta endotoxins [24].

### 2.3. Gammaproteobacteria

#### 2.3.1. *Photorhabdus* spp. and *Xenorhabdus* spp.

The entomopathogenic members of the genera *Photorhabdus* and *Xenorhabdus* are represented by endosymbionts of insecticidal nematodes. The first are typically associated with entomopathogenic nematodes in the genus *Heterorhabditis*, while the second to *Steinernema* species. The pathogenic action usually involves the release of symbiotic bacteria in the insect hemocoel once the nematodes have actively entered the insect body. Here the bacteria proliferate producing various antimicrobial compounds to contrast the growth of other microorganisms. They also release different enzymes that contribute to the degradation processes in the hemocoel, thus creating an ideal environment for the development of the nematode population.

A variety of bacterial virulence factors are involved in the interaction with the susceptible host.

Different *Photorhabdus* and *Xenorhabdus* species producing an insecticidal toxins complex (Tc) have high potential for pest management [25]. Generally, the Tcs are high-molecular weight and multi-subunit proteins that include three components, A, B and C, orally active against different insects [26]. While the mode of action is not completely understood, all these components are normally needed to achieve full toxicity [27].

Another example of insecticidal proteins produced by these bacterial species is represented by the *Photorhabdus* insect related (Pir) proteins, produced by *P. luminescens* (Thomas and Poinar), that show similarity to *B. thuringiensis* delta-endotoxins and have been proposed to be mimics of the juvenile hormone esterases (JHEs) interfering with insect development regulation [28].

In addition to insecticidal toxins and various metabolites, these bacterial endosymbionts have evolved different mechanisms to face the insect immune response. For instance, it has been shown that certain *Photorhabdus* species exploit lipopolysaccharide modifications to resist the action of insect antimicrobial peptides (AMPs) [29], while *X. nematophila* (Poinar and Thomas) interferes with the expression mechanisms of host AMPs [30].

In addition to *P. luminescens* and *X. nematophila*, most studied species include *P. asymbiotica* Fischer-Le Saux *et al.*, *P.temperata* Fischer-Le Saux *et al.*, *X. beddingii* (Akhurst) Akhurst and Boemare, *X. bovienii* (Akhurst) Akhurst and Boemare, *X. japonica* Nishimura, and *X. poinarii* (Akhurst) Akhurst and Boemare. Besides, new species are continuously being identified and characterized [31].

So far, the commercial use of these bacterial endosymbionts is related to the employment of the nematode species with which they are associated.

#### 2.3.2. *Serratia* spp.

The association of *Serratia* spp. with insects or with entomopathogenic nematodes is well documented [32,33,34]. Different species in this genus produce a variety of virulence factors [35]. Common is the production of toxin complexes analogous to those produced by *Xenorhabdus* spp. and *Photorhabdus* spp. *S. entomophila* Grimont *et al.*, a pathogen of the grass grub, *Costelytra zealandica* (White) (Coleoptera: Scarabaeidae) [36], produces Sep proteins (SepA, SepB, SepC), a group of insecticidal toxins showing similarities to the insecticidal toxins of *P. luminescens* [37].

On the other hand, the recent genome sequencing of *S. nematodiphila* Zhang *et al.* also highlighted other pathogenic factors of *Serratia* species. Among these was a variety of secreted extracellular enzymes such as proteases, lipases, and chitinases [38].

It has recently been demonstrated that the pathogenicity of *S. marcescens* Bizio is increased by the action of a serralysin metalloprotease it secretes and that this bacterium is able to suppress cellular immunity by decreasing the adhesive properties of immunosurveillance cells of the insect host [39].

#### 2.3.3. *Yersinia entomophaga*

Isolated from the New Zealand grass grub, *C. zealandica*, *Y. entomophaga* is a non-spore-forming entomopathogenic bacterium characterized by the production of an insecticidal toxin complex (Yen-Tc) showing similarity to those produced by *Photorabdus* spp. [40]. These complexes include three Yen protein families, A, B and C, and two chitinases (Chi1 and Chi2) [41].

The broad insecticidal range of these toxins and their post-ingestion histopathological action in the insect midgut epithelium have been reported [42].

Promising are the studies conducted in field conditions with insecticidal formulations containing *Y. entomophaga* against the pasture pest porina (*Wiseana* spp. larvae) [43].

#### 2.3.4. *Pseudomonas entomophila*

*P. entomophila* is a ubiquitous bacterium that orally infects larvae of insects in different orders determining extensive gut cell damages. Host-pathogen interactions have been studied in experiments with *Drosophila melanogaster* Meigen (Diptera: Drosophilidae), which highlighted a specific post-ingestion immune response [44]. Recent complete genome sequencing of *P. entomophila* revealed a specific secretion system and the associated toxins probably responsible for its entompathogenic properties [45].

### 2.4. Betaproteobacteria

#### 2.4.1. *Burkholderia* spp.

Different insect species harbor symbiotic bacteria of the genus *Burkholderia*, mostly in association withspecific gut regions [46,47]. In addition to these mutualistic relationships with insects, *Burkholderia* sp. has recently been reported to affect ovipositon and fecundity of the bean bug *Riptortus pedestris* (Fabricius) (Hemiptera: Alydidae) [48]. The pontential of *Burkholderia* species as biocontrol agents against different plant pathogens has also been reported [49,50]. More recently, the insecticidal properties of a new strain isolated in soil from Japan and identified as *B. rinojensis* sp. nov., were discovered [51]. Whole cell broth cultures of this bacterial strain, named A396, show oral toxicity and contact effects against the beet armyworm *Spodoptera exigua* Hübner (Lepidoptera: Noctuidae) and the two-spotted spider mite *Tetranychus urticae* Koch (Acari: Tetranychidae). Insecticidal and miticidal properties are maintained after heat-treatment, hence commercial formulations against a variety of chewing and sucking insects and mites are based on heat-killed cells and spent fermentation media. Different bacterial metabolites might be involved in the insecticidal action. Interestingly, a recent study highlighted the biochemical properties of natural compounds produced by fermentation of a *Burkholderia* sp. strain showing high homology to *B. rinojensis* [52].

#### 2.4.2. *Chromobacterium* spp.

A strain of *Chromobacterium subtsugae* Martin *et al.*, isolated from a soil sample in Maryland (USA) and named PRAA4-1T, was discovered to show high insecticidal activity against insect species in different orders, including the Diamondback moth *Plutella xylostella* L. (Lepidoptera: Plutellidae), the Sweet potato whitefly *Bemisia tabaci* Gennadium (Rhynchota: Aleyrodidae**)**, the Southern green stink bug *Nezara viridula* L. (Rhynchota: Pentatomidae), the Southern corn rootworm *Diabrotica undecimpunctata* Mannerheim (Coleoptera*:*Chrysomelidae spectrum), the Western corn rootworm *Diabrotica virgifera* Le Conte (Coleoptera: Chrysomelidae), the Colorado potato beetle *Leptinotarsa decemlineata* Say (Coleoptera*:* Chrysomelidae), and the Small hive beetle *Aethina tumida* Murray (Coleoptera: Nitidulidae) [53,54]. The broad spectrum activity of this strain is related to multiple modes of action probably involving different chemical compounds produced by the bacterium. Among the bacterial metabolites, *C. subtsugae* synthesizes the tryptophan derivative violacein, which confers a typical violet color to its colonies. In addition to this, various molecules produced by this species have been characterized and associated with the insecticidal action [55]. Bioactive compounds were reported to be associated to the stationary growth phase [56], and the heat-stability of insecticidal toxins was also demonstrated [57]. The active ingredient of available commercial formulations is represented by *C. subtsugae* strain PRAA4-1T and spent fermentation media.

More recently, a new strain identified as *Chromobacterium* (Csp_P), isolated from the midgut of *Aedes aegypti* L. (Diptera: Culicidae) was shown to be able to colonize the insect midgut and to display entomopathogenic and anti-pathogen properties [58].

### 2.5. Actinobacteria

#### 2.5.1. *Streptomyces* spp.

Different *Streptomyces* spp. are associated with herbivorous insects that take advantage of their cellulolytic properties [59]. Other species and strains in this genus produce a variety of metabolites acting as potent toxins against either phytopathogenic microbials or insect pests [60]. Among the first discovered insecticidal substances produced by *Streptomyces* species are flavensomycin [61], antimycin A [62], piericidins [63], macrotetralides [64] and prasinons [65]. Later on, the insecticidal and anthelmintic activities of avermectins produced by the soil Actinomycete *Streptomyces avermitilis* MS & Dwas, were discovered [66]. These macrocyclic lactone derivatives target the gamma-aminobutyric acid (GABA) receptor in the insect peripheral nervous system. The enhancement of GABA binding generates a cascade of events resulting in the inhibition of neurotransmission and paralysis of the neuromuscular systems [67].

Insecticides based on avermectins include a mixture of avermectin B1a and avermectin B1b, known as abamectin, that act by contact and ingestion and have a limited plant translaminar activity. Analogous substances produced by *Streptomyces* species include Emamectin, especially toxic to Lepidoptera, and Milbemectin, isolated from *S. hygroscopicus* Jensen. A variety of other secondary metabolites produced by diverse *Streptomyces* species, have been isolated and characterized so far. Commercialized products have been very successful against ectoparasites and endoparasites with medical and veterinary importance.

#### 2.5.2. *Saccharopolyspora spinosa*

*Saccharopolyspora spinosa* Mertz and Yao was discovered during a screening program where the insecticidal activity of the isolate A83543 emerged [68]. Subsequent assays highlighted the broad toxicity of specific compounds isolated from the fermentation broth that were given the generic name of spynosins. The major component is spinosyn A, whose structure comprises a tetracyclic polyketide aglycone to which a neutral saccharide substituent is attached [69]. A variety of spinosyn analogs have been isolated and many studies have been conducted to investigate the pathway of spinosyn biosynthesis, which led to the characterization of specific gene clusters [70].

Since the first experimentations, spinosyns exhibited broad-spectrum activity against insect species in different orders, especially Lepidoptera and Diptera [71]. The natural *S. spinosa* fermentation-derived mixtures were named “spinosad” and contain spinosyn A and spinosyn D, as major and minor component, respectively. The biological activity of numerous semisynthetic derivatives has been studied [72].

The insecticidal mode of action of spinosyns is not completely understood, but is considered unique in comparison with other insecticides. It has been demonstrated their interaction with g-aminobutyric acid receptors and nicotinic acetylcholine receptors, eventually leading to the disruption of neuronal activity and consequent insect paralysis and death [73]. Despite their broad spectrum of activity against insects, spinosyns are associated with a low risk toward non-target species, including mammals and various aquatic organisms, in comparison with other insecticides [74].

The continuous research and industrial interest in this field has recently led to the discovery of a variety of spinosyn-related compounds produced by another *Saccharopolyspora* species, *S. pogona* [75].

## 3. Use of Entomopathogenic Bacteria in Integrated Pest Management

Insect pathogenic bacteria and their derived products represent the active substances of various “biopesticides”. There is a range of definitions for this term, but it essentially includes mass reared living organisms (natural insect predators and parasitoids), nematodes and micro-organisms (bacteria, fungi, microsporidia, virus), natural compounds from plant extracts, and semiochemicals (e.g., insect pheromones). Besides, in countries where their use is permitted, biopesticides comprise genetically modified plants that express genes conferring resistance against pests or diseases (plant incorporated products).

Biological control agents, like bacterial entomopathogens, are generally recognized as lower risk substances than conventional chemical pesticides, and various benefits are associated with their use. For instance, their mode of action is normally more complex than conventional chemicals, targeting a diversity of action sites, which makes the development of resistant pests less likely. Although entomopathogenic bacteria can be used as stand-alone products for pest management in organic farming, their use in rotation or combination with chemicals is strongly encouraged to achieve full efficacy and eco-sustainability. Many studies have highlighted compatibility and synergistic effects of entomopathogenic bacteria and chemical substances [76,77,78]. Among the other advantages of including biopesticides in pest management programs are their safety for workers, the reduction of residues on crop and the flexibility on harvests, due to minimal or no pre-harvest interval.

The effectiveness of entomopathogenic bacteria is often associated with a proper application in the field. For instance, in the case of products that act by ingestion (*i.e.*, *Bt* based products), timing is critical to ensure that the bacterial toxins remain stable in the environment until they are ingested by their target insect stage [7]. Another aspect is to ensure a proper coverage of substrates (e.g., foliage) frequented or eaten by insects. This has led to the development of special processing and formulation of bacteria-based bioinsecticides, with the aim of maximizing shelf-life, improving dispersion and adhesion, reducing spray drift and above all enhancing efficacy. A variety of adjuvants and additives for microbial formulations have been developed by the industry. These include dispersants, surfactants, wetters, spreaders, drift control agents, pH buffers, antifoam agents, carriers, phagostimulants and attractants [79]. Depending on the application target and on the adverse environment conditions, a choice of solid and liquid formulations is available. The first include dusts, granules, briquettes and wettable powders (WPs), while liquid suspensions may consist of suspension concentrates (SCs), emulsions and encapsulations. Advanced technologies aiming at increasing residual effects comprise microencapsulations and microgranules.

The variety of entomopathogenic bacteria based active substances authorized for commercialization and of their relative commercial products is significantly increasing worldwide. Consequently, their market is expanding into new segments, thus broadening their use for pest management, traditionally relegated to niche contexts (forest, public health, protected crops) [6].

Besides optimizing efficacy, modern pest management strategies tend to minimize the impact on the environment and on non-target organisms. This is also in line with the need to meet current regulations on the maximum residue levels (MRLs) for synthetic pesticides. The management of pest counter-adaptation (resistance) to pesticidal products is another concern in the agro-ecosystem. The integration of bio-based pesticides in pest management programs in many cases represents an important resource to face these challenges. During the last years, the number of microbial products available has grown significantly and many efforts are dedicated to increasing the awareness and to fostering the adoption of biopesticides in integrated pest management programs. This includes the implementation of worldwide industrial understandings like the Biopesticide Industry Alliance (BPIA) and the International Biocontrol Manufacturers’ Association (IBMA). According to this trend, a database of available biopesticides is maintained within the context of the biopesticide program of the Interregional Research Project Number 4 (IR-4) at Rutgers University (U.S.).

Many biopesticides have recently gained interest within the legislative framework of most important regions like USA and Europe, fostering the use of low risk active substances in agriculture. In the United States, for the pre-market approval (registration) of any pesticide, the Federal Insecticide, Fungicide, and Rodenticide Act (FIFRA) requires a specific evaluation by the Environment Protection Agency (EPA). Since 1994, to facilitate the registration of biopesticides a Biopesticides and Pollution Prevention Division was established in the Office of Pesticide Programs. In Europe, to facilitate procedures for registration of new products, previously based on EU Directive 91/414, new criteria have recently been implemented by Regulation (EC) No 1107/2009. Besides, specific data requirements and the principles for evaluation and authorization of new plant protection products (PPPs) have been established by recent Regulation (EU) No 283 and 284/2013, and Regulation (EU) No 546/2011, respectively. On this basis, any new active substance is first authorized at the EU level, while the formulated product is subjected to approval at Member State level.

## 4. Market Overview

Based on a recent report published by BCC Research LLC (CHM029E, June 2014), the global market for pesticides, including both the synthetic pesticides and the biopesticides segments, is estimated to grow at a compound annual growth rate (CAGR) of 6.3% and to reach more than $80 billion by 2019.

The fastest growing segment of this market is represented by biopesticides (around 4%) that are expected to increase at more than twice the rate of synthetic pesticides. This segment comprises both biochemical (*i.e.*, semiochemicals, plant extracts) and microbial biopesticides. Among the latter are included products based on bacteria, fungi, virus and nematodes, targeting different kinds of plant pests and parasites. A different category is represented by biocides, including products for public health like microbials targeting mosquitoes and flies, whose commercialization is differently regulated. This market is also expected to experience substantial growth in future (Research and Markets, ID: 2692355, October 2013).

The increased industrial interest in biopesticides is shaping a changing global scenario featured by strategic partnerships, mergers and acquisitions of companies. Significant examples of this trend are the recent acquisitions of the US-based biological company AgraQuest and of the German company Prophyta GmbH by Bayer CropScience. Similarly, BASF acquired Becker Underwood Inc., while Syngenta agreed to acquire the US-based biotechnology company Pasteuria Bioscience Inc. and the Swiss Devgen. On the other side, Monsanto and Novozymes have established an historical BioAg Alliance committed to the discovery and development of new microbial solutions for agriculture.

## 5. Conclusions

The need to feed a growing human population requires continuous advancements in pest management systems limiting production losses in major crops [80]. On the other side, the land available for cultivation and farming on Earth is limited, which requires the development of new technologies supporting improvements in productivity. Safeguarding the environment and human health, and the need to manage the development of insect resistance to pesticides, are additional concerns. For all these reasons, the integration of bio-based insecticides in combination or rotation with synthetic formulations is strongly recommended. This is in line with the expectations of fruit and vegetable consumers and with the requirements of a modern legislative framework on the use of IPM in agriculture.

Following this trend, an increasing academic and industrial interest in the discovery and development of new bioinsecticides is being experienced by the scientific community working in the field of insect pathology. The relative market segments and the industrial interest in this field are also growing at a significant rate. As a result, researches on entomopathogenic bacteria are gaining momentum and new discoveries are expected in the near future. Due to the regulatory issues related to the pre-market authorization of new active substances, the availability of bio-based products, including entomopathogenic bacteria, is still limited to certain crop pests. However, both research funding bodies and industry are progressing in the direction of increasing investments in this field, which will result in a continuous expansion of the repertoire of insect pathogenic bacteria-based formulations available for integrated pest management.
